# The Temporal Dynamics of Neighborhood Disadvantage in Childhood and Subsequent Problem Behavior in Adolescence

**DOI:** 10.1007/s10964-018-0878-6

**Published:** 2018-06-30

**Authors:** Tom Kleinepier, Maarten van Ham

**Affiliations:** 1 OTB—Research for the Built Environment, Faculty of Architecture and the Built Environment, Delft University of Technology, PO Box 5030, 2600 GA Delft, The Netherlands; 2 School of Geography and Geoscience, University of St Andrews, Irvine Building, North Street, St Andrews, Fife, KY16 9AL Scotland

**Keywords:** Neighborhood effects, Temporal dynamics, Childhood, Adolescence, Problem behavior, Sequence analysis

## Abstract

Research on neighborhood effects has increasingly focused on how long children have lived in a deprived neighborhood during childhood (duration), but has typically ignored when in childhood the exposure occurred (timing) and whether neighborhood circumstances were improving or deteriorating (sequencing). In this article, the authors applied sequence analysis to simultaneously capture children’s duration, timing, and sequencing of exposure to neighborhood (dis)advantage in childhood. Logistic regression analysis was subsequently used to test how different patterns of exposure are related to teenage parenthood, school dropout, and delinquent behavior. Using register data from the Netherlands, an entire cohort was followed from birth in 1995 up until age 19 in 2014 (*N* = 168,645, 48.8% females, 83.2% native Dutch). Compared to children who had lived in a deprived neighborhood throughout childhood, children who were exposed to neighborhood deprivation only during adolescence were found to be equally likely to become a teenage parent and were even more likely to drop out of school. Unexpectedly, children who had lived in an affluent neighborhood throughout childhood were most likely to engage in delinquent behavior. Possible explanations and implications are discussed.

## Introduction

It has repeatedly been shown that children who grow up in a deprived neighborhood are more likely to engage in several types of problem behavior during adolescence than children who grow up in more affluent neighborhoods (Leventhal and Brooks-Gunn 2000). For example, previous studies have found neighborhood effects on high school dropout rates (Wodtke et al. 2011), juvenile delinquency (Damm and Dustmann 2014), adolescent substance use (Kulis et al. 2007), and teenage childbearing (South and Crowder 2010). Yet, while there is extensive empirical evidence supporting the existence of neighborhood effects on adolescent development and behavior, a recurring issue is that the estimated effects are often relatively weak. Moreover, when family socioeconomic status and school contextual variables are controlled for, the estimated impact of the residential neighborhood often becomes even smaller (Nieuwenhuis and Hooimeijer 2016). This has led several researchers to be sceptical about the importance of the neighborhood context in shaping young people’s life perspectives (Cheshire 2012).

One reason for why in general only weak neighborhood effects are observed could be that previous research has often neglected or not adequately addressed the temporal dynamics of children’s neighborhood context (Sharkey and Faber 2014). Until recently, research has almost exclusively relied on single point-in-time indicators of children’s neighborhood characteristics (e.g., Brooks-Gunn et al. 1993). These measures have been criticized for the fact that children’s neighborhood characteristics may change over time, either because families move to a different neighborhood or because neighborhoods themselves change over time (Kleinepier and van Ham 2017). The increasing availability of longitudinal data has enabled recent studies to develop more dynamic measures of children’s neighborhood experiences. Most of these studies have focused on the *duration* of exposure to poor and nonpoor neighborhoods during childhood, showing that measures of cumulative exposure exert a stronger effect on outcomes in later life than point-in-time measures of neighborhood disadvantage (e.g., Wodtke et al. 2011).

However, while researchers have increasingly focused on the amount of time children spend living in poverty neighborhoods during childhood, little attention has been paid to examining whether the *timing* of exposure (e.g., early childhood vs. adolescence) to neighborhood (dis)advantage has differential effects on adolescent outcomes. At the same time, both theory and empirical research suggest that the consequences of living in a deprived neighborhood may vary across different developmental periods in childhood (Wodtke 2013). The few prior studies that have investigated timing effects of neighborhood (dis)advantage have typically estimated the effect of neighborhood deprivation at one stage in childhood on some dependent variable while controlling for neighborhood deprivation at other stages. A disadvantage of this approach is that it does not differentiate between children who live in a poor neighborhood throughout childhood, those who move into poor neighborhoods, those who move out of poor neighborhoods, and those who move in and out of poor neighborhoods. In other words, these studies do not take into account the *sequencing* of exposure to neighborhood deprivation during childhood. Although results are somewhat inconsistent, several studies suggest that moving to a poorer neighborhood during childhood is related to problem behavior of youth, highlighting the need to focus on sequencing of neighborhood poverty during childhood as well (Nieuwenhuis et al. 2017).

In this article, sequence analysis is applied to simultaneously capture children’s duration, timing, and sequencing of exposure to neighborhood disadvantage during childhood. While sequence analysis has increasingly been used in the field of neighborhood studies, its application has so far mainly remained limited to visualization purposes (for an exception, see Kleinepier et al. 2018). We go one step further and use optimal matching followed by cluster analysis to empirically categorize children into a limited number of groups on the basis of similarities in terms of duration, timing, and sequencing of exposure to neighborhood deprivation. The primary aim of this study is to examine how and to what extent such different patterns of exposure to neighborhood disadvantage during childhood are related to three types of problem behavior in adolescence, viz. teenage childbearing, school dropout, and delinquent behavior.

### Neighborhood Effects

**Fig. 1:**
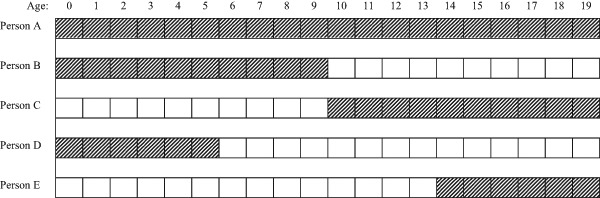
A hypothetical example of five different patterns of exposure to neighborhood deprivation (as indicated by shading) in childhood

Traditionally, studies linking neighborhood disadvantage during childhood to outcomes in later life have investigated the effects of single point-in-time measurements of neighborhood context on individual outcomes. For example, Crane 1991 defines teenagers’ neighborhood environments based on their places of residence in 1970. Likewise, Brooks-Gunn et al. 1993 and Sucoff and Upchurch 1998 measure children’s neighborhood characteristics at age 14. Viewed from a life-course perspective (Elder 1998), however, children are likely to not only affected by their current residential location, but also by their past neighborhood experiences. It has been shown that there is substantial variation over time in children’s neighborhood characteristics, particularly among those who moved home (Kleinepier and van Ham 2017). Researchers have therefore started to develop more dynamic conceptualizations of children’s neighborhood environments (i.e., duration, timing, and sequencing of exposure). These different approaches are explained with a hypothetical example of five children who experience distinctive patterns of exposure to neighborhood deprivation (as indicated by shading) in Fig. 1. However, before discussing these in greater detail, it is worth outlining the main mechanisms of neighborhood effects because they help explaining why the magnitude of these effects depends on duration, timing, and sequencing of exposure.

Many potential causal mechanisms for the explanation of neighborhood effects have been proposed in the literature. Galster 2012 grouped these into four categories: social-interactive, environmental, institutional, and geographical mechanisms. Social-interactive mechanisms assume that neighborhood effects transpire because the population composition of the residential neighborhood affects with whom people interact. For example, children in disadvantaged neighborhoods may, through contact with peers who are neighbors, be exposed to less favorable attitudes toward the labor market, deviant behavior, the value of education, and so on (Ingoldsby et al. 2006). Moreover, adults in deprived neighborhoods may provide negative role models to children and assert lower levels of social control than adults in more affluent neighborhoods (Beyers et al. 2003). Environmental mechanisms focus on the physical condition of neighborhoods (e.g., litter, graffiti, air pollution) that may affect directly the physical health or behavior of residents. Indeed, Caughy et al. 2008 found that children who live in neighborhoods with high degrees of physical and social disorder tend to have greater internalizing problems. Finally, institutional and geographical mechanisms are concerned with the influence of institutions and organizations within and near the neighborhood respectively, such as day care facilities, high-quality schools, and medical clinics. The relative lack of such institutions in or near deprived neighborhoods may adversely affect children’s behavior (Gaias et al. 2017).

### Duration of Exposure

At a general level, the life course perspective recognizes the importance of the cumulative impact of experiences during childhood on children’s behaviors and achievement in later life (Elder 1998). Various studies in recent years have picked up on this notion and have focused on the duration of exposure to poverty neighborhoods during childhood, often referred to as “cumulative exposure” models (Anderson et al. 2014). The assumption here is that children who are consistently exposed to neighborhood deprivation may be influenced more than those who experience socio-spatial disadvantage for only a short period of time. This can be linked to the social-interactive mechanisms of neighborhood effects outlined earlier. For example, it could be argued that a long stay in a neighborhood where social norms prevail which are more accepting of early childbearing, devalue education, and condone crime, leads to higher rates of teenage parenthood, school dropout, or delinquent behavior (Friedrichs and Blasius 2003). In contrast, a brief period in such a neighborhood is likely not enough for the local values and behaviors to become internalized. Likewise, the cumulative risk that children engage in problem behaviors increases when they spend more time in neighborhoods that lack adequate supervision (Wodtke et al. 2011). Regarding environmental mechanisms of neighborhood effects, the extent to which individuals are harmed by the physical conditions of a neighborhood (e.g., air pollution) strongly depends on the time spent in the neighborhood (Schwartz 2006). Thus, there are multiple reasons to account for the length of residence in disadvantaged neighborhoods during childhood.

In line with this reasoning, empirical research showed that measuring duration of exposure to neighborhood poverty during childhood yields stronger effects than single point-in-time measures of neighborhood disadvantage on externalizing and internalizing problems (Wheaton and Clarke 2003), high-school dropout (Crowder and South 2011), and teenage premarital childbearing (South and Crowder 2010). As regards the example in Fig. 1, if it is indeed primarily the duration of exposure to neighborhood disadvantage driving problem behavior among youth, one would expect Person A to be affected strongest by neighborhood disadvantage because it lasts longest (i.e., throughout the entire childhood life course) and Person D and E to suffer the least consequences from their neighborhood deprivation.

### Timing of Exposure

Importantly, however, by solely focusing on the duration of exposure to neighborhood disadvantage during childhood, one would, for example, fail to recognize the obvious difference between Person B and C—both have lived in a deprived neighborhood for a duration of 10 years, but never at the same age (Fig. 1). This brings us to another key component of the life-course perspective: the timing of lives. This notion emphasizes that the impact of people’s environment on subsequent behavior is contingent on *when* the exposure occurs in a person’s life (Elder 1998). Yet, so far only a few studies have paid attention to whether the consequences of living in a deprived neighborhood depend on the developmental timing of exposure in childhood. As a result, the literature on neighborhood effects does not offer clear guidance on this issue. Based on different theoretical perspectives, two competing lines of reasoning can be put forward.

The first line of reasoning argues that neighborhood disadvantage is particularly detrimental if experienced early in the childhood life course. This argument stems from developmental and brain research pointing to early childhood as the key period for cognitive development (Heckman 2006) and a period of unique vulnerability to environmental influences (Anderson et al. 2014). The timing principle of the life-course perspective highlights that such initial disadvantage may accumulate over time, generating further disadvantage (Elder 1998). Although it is not fully clear how neighborhood effects operate in early childhood, it could be argued that social-interactive mechanisms are less important because children’s environments are more controlled by parents during this period (Kiesner et al. 2003). Yet, neighborhoods can influence young children indirectly by affecting their parents and families. For example, parents in deprived neighborhoods experience higher levels of stress which, in turn, increases children’s externalizing problems (Plybon and Kliewer 2001). Regarding environmental mechanisms of neighborhood effects, the developmental perspectives reviewed here further suggest that exposure to poor physical conditions of a neighborhood may be most harmful during early childhood. From the perspective of institutional and geographical mechanisms, neighborhood disadvantage in early childhood could be detrimental due to lower-quality child care centers, kindergartens, and playgrounds. In addition, children who are born in deprived neighborhoods are more likely to attend underequipped primary schools, placing them on disadvantaged life-course trajectories that may eventually lead to problem behavior in adolescence.

In somewhat indirect support of the importance of neighborhood context during early childhood, McCulloch and Joshi 2001 find that neighborhood poverty has a strong negative effect on test scores for children aged 4–5 years, a relatively weak effect for children aged 6–9 years, and no effect among children aged between 10–18 years. Wheaton and Clarke 2003 showed that exposure to neighborhood poverty in early childhood had larger effects on mental health in early adulthood than neighborhood disadvantage in adolescence or early adulthood. Finally, a study by Anderson et al. 2014 suggests that living in a neighborhood with more affluent residents in early childhood, but not adolescence, is associated with higher reading abilities in adolescence. Thus, several studies suggest that exposure to neighborhood disadvantage in early childhood, as opposed to other developmental periods in childhood, is most important for educational achievement and problem behavior of youth. If so, one would expect Person A, B, and D in Fig. 1 to be affected strongest by neighborhood deprivation.

The second line of reasoning argues that neighborhood deprivation has stronger effects if it is experienced later in childhood (i.e., in adolescence). This argument is primarily based on social-interactive mechanisms of neighborhood effects. Parents grant more autonomy to their children as they grow older, implying that adolescents have greater exposure to extrafamilial influences, including peers in the neighborhood (Prinstein and Dodge 2008). Indeed, the influences of peers on problem behavior have been found to be particularly strong during adolescence, likely because of increases in the amount of time spent with peers, the importance of peer relationships, and greater susceptibility to peer influences (Prinstein and Dodge 2008). Ingoldsby et al. 2006 suggest that effect of deviant peers in the neighborhood on children’s antisocial behavior is stronger later in childhood than in early childhood. Specifically with regard to the risk of teenage parenthood, a person’s peer group during the teenage years might be more important than the peer group in early childhood because it is biologically (almost) impossible to have children at younger ages. Furthermore, adolescents are more aware of their potentially disadvantaged circumstances than young children which, in turn, might lower their academic aspirations and increase the risk of high school dropout (Wagmiller et al. 2006). Finally, the residential setting may be more salient in the adolescence than earlier in childhood from the perspective of institutional mechanisms as well. Leventhal and Brooks-Gunn 2000 indicate that adolescents are more engaged with institutions such as schools and the police than young children.

Consistent with this emphasis on exposure to neighborhood deprivation late in childhood, previous research showed that exposure to neighborhood poverty during adolescence has a stronger negative effect on high school graduation (Wodtke et al. 2016) and a more positive effect on teenage childbearing (Wodtke 2013) than exposure earlier during childhood. In view of these theoretical propositions and empirical findings, one would expect that Person A, C, and E are harmed most by neighborhood deprivation (Fig. 1).

### Sequencing of Exposure

Yet another line of argumentation emphasizes changes in neighborhood circumstances over time, i.e. the sequencing of neighborhood deprivation. From a life course perspective, a change in neighborhood circumstances during childhood may be considered a “turning point” in children’s lives (Elder 1998). Turning points can be positive or negative in nature. Moving from an affluent neighborhood towards a deprived neighborhood may be regarded as a negative turning point. In fact, there are reasons to expect that children who experience stable but disadvantaged neighborhood circumstances are affected less negatively than children whose neighborhood circumstances worsen. For example, while children who have always lived in a poor neighborhood might be accustomed to living in such areas and adjust their lives accordingly, children moving into poverty neighborhoods may be overwhelmed by the new residential environment which might lead to problem behavior and lower educational attainment (Moore et al. 2002). Intuitively, moving from a poor neighborhood to a low-poverty neighborhood can be seen as a positive turning point, because the new neighborhood provides more positive role models (social-interactive mechanisms), improved physical conditions (environmental mechanisms), and access to higher-quality schools (institutional and geographical mechanisms). However, moving to a more affluent neighborhood may change the family’s relative economic position compared to their neighbors. According to relative deprivation theory, this relatively lower position in the social hierarchy can be a source of dissatisfaction (Galster 2012).

Most studies on the sequencing of neighborhood deprivation have been concerned with upward neighborhood mobility. For instance, the Moving To Opportunity program in the US sought to relocate poor families out of high-poverty neighborhoods by providing housing vouchers. Studies on this experiment portray a rather mixed picture, with some finding positive effects (Chetty et al. 2016) some finding negative effects (Kling et al. 2005), and still others finding no effects at all (Ludwig et al. 2013) on adolescents’ schooling and behavioral outcomes. Importantly, Chetty et al. 2016 showed that moving to low-poverty neighborhoods before age 13 increases college attendance and reduces single parenthood rates, while children who moved after age 13 were negatively affected by the treatment. The decline in the gains from moving to a more affluent neighborhood with age may be related to differences in timing and duration of exposure to low-poverty neighborhoods. Indeed, children who moved late in childhood were only exposed to more affluent neighborhoods during adolescence and for shorter durations than children who moved earlier in childhood. Chetty et al. 2016 therefore argue that one reason for inconsistent findings across studies may be the pooling of younger and older children in previous research on the Moving To Opportunity experiment.

Furthermore, a recent study using Dutch data showed that adolescents who moved to a more affluent neighborhood are more likely to have increased levels of depression, social phobia, aggression, and conflict with their parents (Nieuwenhuis et al. 2017). The authors interpret this as potential evidence for the relative deprivation hypothesis. Other research in the UK, however, showed that downward movers had more mental health problems than those moving upward in neighborhood hierarchy (Tunstall et al. 2012). The latter two studies are difficult to compare directly because they are conducted in different countries, measure neighborhood deprivation in different ways, and use different outcome variables. Nevertheless, these contrasting findings highlight the lack of consensus concerning the sequencing of neighborhood deprivation. In terms of the example in Fig. 1, if as some studies suggest that moving into poverty neighborhoods is most harmful for child development, one would expect Person C and E to be most likely to engage in problem behavior. However, if moving to a more affluent neighborhood leads to problematic behavior as other studies suggest, then Person B and D are in the least favorable position.

## Current Study

The temporal dimension of neighborhood effects on children’s outcomes has received increased empirical attention in recent years (Sharkey and Faber 2014). Researchers generally agree now that prolonged exposure to poverty neighborhoods has a stronger negative effect on individual outcomes than does brief exposure. However, the neighborhood-effects literature is much smaller and less consistent regarding the timing and sequencing of exposure. Moreover, the relative importance of how long (duration), when (timing), and in what order (sequencing) children are exposed to neighborhood deprivation remains to be explored. The current study aims to address these issues in two ways. First, techniques of sequence analysis are used to simultaneously capture the duration, timing, and sequencing of exposure to neighborhood deprivation in childhood. More precisely, optimal matching followed by cluster analysis is applied to categorize children into a limited number of groups covering different patterns of exposure to neighborhood (dis)advantage during childhood. The clusters are then used to predict different types of adolescent problem behavior, allowing for a direct test of the competing theoretical hypotheses outlined earlier (cf. Wagmiller et al. 2006). Second, because an important reason for inconsistent findings across studies may be the use of different outcome variables in these studies, this study focuses on three commonly studied types of adolescent problem behavior: teenage parenthood, school dropout, and delinquent behavior. In other words, the use of three different outcome variables allows us to examine whether different patterns of exposure to neighborhood (dis)advantage in childhood correspond differently to different types of problem behavior in adolescence.

## Methods

### Sample

This study uses longitudinal administrative microdata derived from the Dutch population registers: the System of Social statistical Datasets (SSD; Bakker et al. 2014). The SSD, hosted by Statistics Netherlands, allows for combining data from various administrative sources for statistical purposes. The different administrative registers provide a wide range of socioeconomic and demographic information on every legal inhabitant of the Netherlands. Examples of these registers include the municipal population register (e.g., ethnic origin, marital status, age), tax registers (e.g., income, employment), and educational registers (e.g., educational level, school enrolment). In addition, the data are geo-referenced, indicating the residential neighborhood of each individual at different spatial scales (e.g., 100 × 100 and 500 × 500 meter grids). Data were available for the period 1995–2014. We selected all children who were born in the Netherlands in 1995 and followed them from birth (in 1995) up until age 19 (in 2014). Children who themselves and/or whose both parents died or emigrated during the observation period were excluded from the analysis (*N* = 23,107; 12%).^1^ This leaves us with a total research population of 168,645 children.

### Measures

We use three dependent variables covering different types of problem behavior: teenage parenthood, school dropout, and delinquent behavior. Table 1 shows the percentage of children who exhibited each of the three types of problem behavior across ages 12–19 (none of the problem behaviors were observed before age 12). In line with previous research (Prinstein and Dodge 2008), problem behavior is most prevalent during late adolescence. Because only very few children engaged in problem behavior more than once, all three dependent variables were coded as dichotomous variables indicating whether or not the child has engaged in the problem behavior. Table 2 provides descriptive statistics for the variables described below.

**Table 1:** Percentage distribution of the three problem behavior variables across ages 12–19

Age	Teenage parenthood		School dropout		Delinquent behavior	
	%	Cumulative %	%	Cumulative %	%	Cumulative %
12	0.00	0.00	0.02	0.02	0.02	0.02
13	0.00	0.00	0.04	0.06	1.01	1.03
14	0.00	0.00	1.00	1.06	1.75	2.78
15	0.01	0.01	1.00	2.06	1.78	4.56
16	0.03	0.04	1.49	3.55	1.79	6.35
17	0.08	0.12	3.70	7.25	2.44	8.79
18	0.18	0.29	3.28	10.53	0.00	8.79
19	0.69	0.98	2.12	12.65	0.00	8.79

Source: System of Social statistical Datasets (SSD)

#### Teenage Parenthood

Teenage parenthood is defined as having a child before the age of 20 (0 = no, 1 = yes). This was determined through the record linkage of parents and children. The cut-off age of 20 was chosen because the number of individuals who become a parent before age 19 is very low (Table 1). Moreover, this definition of teenage parenthood follows the convention in the literature (e.g., Wodtke 2013).

#### School Dropout

School dropout is measured with a dichotomous variable indicating whether the individual had left education without having obtained a start qualification before the age of 20 (0 = no, 1 = yes). A start qualification is defined as a higher general or pre-university secondary school diploma (“havo” or “vwo” graduate) or an intermediate vocational education diploma (“mbo” level 2 graduate). The information was obtained from the basic register for education (*Basisregister Onderwijs*), in which all students in secondary education in the Netherlands are registered. The measurement moment is the first of October each year. A student is thus considered to have dropped out if he/she does not have a start qualification and is not enrolled in school on October 1, while he/she was in the year before. This means, for example, that a student dropping out in the school year 2006–2007 and returning in education before the first of October 2007 is not registered as a dropout.

#### Delinquent Behavior

Delinquent behavior is measured by assessing whether the child had been sent to “Bureau Halt” (0 = no, 1 = yes). Youth between the ages of 12 and 17 arrested by the police for having committed certain minor offences are referred to Bureau Halt. This organisation then sanctions the children, often involving certain learning and work tasks. If the person carries out the Halt sanction satisfactorily, no further prosecution takes place and no entry is made in the criminal records.

#### Neighborhood Trajectory

The definition of neighborhood (dis)advantage is based on the average annual income in each neighborhood for each year of observation^2^. Specifically, neighborhoods are sorted into quintiles based on the neighborhoods’ average income from the poorest to the wealthiest of tracts. Neighborhoods in the top 20 percent of the income distribution are labelled as “affluent”, the bottom 20 percent as “deprived”, and the remaining 60 percent as “middle-income” neighborhoods. Using this three-fold distinction, a sequence of neighborhood conditions is constructed for each child. Sequence analysis is applied (see the section “Analytic Strategy” for details) to classify all sequences into seven classes of children who experienced similar histories of exposure to neighborhood (dis)advantage in their childhood: (1) consistent deprivation, (2) early deprivation, (3) late deprivation, (4) consistent middle-income, (5) early affluence, (6) late affluence, and (7) consistent affluence.

Neighborhood boundaries were defined using 500 × 500 meter grids (based on geographical coordinates). Prior research showed that children’s neighborhood trajectories are very similar when using 100 × 100 rather than 500 × 500 meter grids to define neighborhoods (Kleinepier et al. 2018). As compared to standard administrative units (e.g., zipcode areas), grid cells have the advantage that they are smaller and therefore more likely to depict inhabitants’ perceived neighborhood environment (Coulton et al. 2013). Moreover, the boundaries of these grid cells remain constant over time, which prohibits that neighborhoods change over time as a result of administrative boundary changes. A disadvantage of grid-defined neighborhoods is that they ignore physical barriers, such as a major highway or river.

#### Ethnicity

The ethnic background of the child is based on the mother’s country of birth or the father’s country of birth in case the mother was born in the Netherlands. Six ethnic minority groups are distinguished: (1) Turkish, (2) Moroccan, (3) Surinamese, (4) Antillean, (5) other non-Western, and (6) Western ethnic minorities. Children with both parents born in the Netherlands are classified as native Dutch and serve as the reference group.

#### Sex

Sex is a dummy variable (0 = female, 1 = male).

#### Educational Level

Educational level of the child is based on the track placement in the first year of secondary school, around the age of 12. The following three categories are distinguished: (1) pre-vocational secondary education (“vmbo”), (2) higher general secondary education (“havo”) and (3) pre-university education (“vwo”).

#### Parental Educational Level

Parental educational level is measured for both parents separately with a dummy variable indicating whether the father/mother obtained a degree in higher education, i.e. bachelor degree or higher (0 = no, 1 = yes). Unfortunately, the SSD provides no information on degrees obtained abroad or before 1986. Therefore, an additional dummy variable was included for cases missing data on parental educational attainment.

#### Parental Labor Force Participation

Parental labor force participation is measured for both parents separately and is based on the period 1999–2014 because data on employment and income were not available for the period 1995–1998. The total number of years the father/mother was employed during the period 1999–2014 was divided by 16 (i.e., total years of observation).

#### Household Income

Household income is measured as the average household income during the years 1999–2014 for reasons of data availability. The household income in each year was first corrected for inflation relative to the base year 1999 and adjusted for household size by dividing the household income in each year by the square root of household size in the given year. This “square-root equivalence scale” presumes that, for example, a household of four persons has financial needs twice as large as a household composed of a single person (OECD 2013). A natural logarithmic specification of the average household income was chosen to account for its right-skewed distribution.

#### Residential Mobility

Residential mobility is measured with a set of dummy variables indicating the number of times the child changed residences during the observation period: (1) no moves, (2) one move, (3) two moves, and (4) three or more moves.

#### Household Size

Household size is a linear variable indicating the number of people living in the same household as the child in 1995 (including the child).

#### Parental Union Status

Parental union status is distinguished into four categories: (1) parents remained together, (2) parents never lived together after child was born, (3) parents divorced, separated, or one parent died during observation period, and (4) parents started living together after initially living apart.

#### Age Difference with Parents

Age difference with parents is measured in years as two continuous variables (for the father and mother separately) (Table 2).

### Analytic Strategy

As explained above, based on quintiles of the neighborhood income distribution in each year of observation, all neighborhoods were classified into three types of neighborhoods: deprived (Q1), middle-income (Q2–Q4), and affluent neighborhoods (Q5). Recall that all children are observed for the entire 20-year study period, i.e. from birth up until 19 years of age. We thus break down each individual’s neighborhood history into a set of 20 discrete time units (one for each age) that can take three possible values: deprived, middle-income, or affluent. The number of possible sequences is very large (3^20^ = 3486,784,401) and raises problems of complexity when comparing all trajectories individually.

We therefore reduce complexity by creating an empirical typology of children’s neighborhood trajectories using sequence analysis. First, distances were computed between all individual sequences using the optimal matching metric (for details, see Abbott and Tsay 2000). This metric measures the dissimilarity of two sequences by considering how much effort must be performed to transform one sequence into the other. In this procedure, there are three operations available: insertion, deletion, and substitution. As an example, consider the sequences *x* [ABCD] and *y* [BCDA]. In metrics which allow only substitution operations (e.g., Hamming distance), transforming sequence *x* into sequence *y* requires four substitution operations (i.e., no overlap). However, both sequences have the subsequence [BCD]. Optimal matching recognizes such similarity that is out of alignment via insertion and deletion. That is, sequence *y* can be transformed into sequence *x* by inserting an “A” at the beginning of the sequence and deleting the “A” at its end (or vice versa). A cost is assigned to each of the three operations by the researcher. The distance between two sequences is defined as the cheapest set of operations that edit one sequence into another. In this study, insertion/deletion costs were set at 1 and substitution costs were defined as the inverse of the transition rates, following the approach used most widely in the literature (e.g., Kleinepier et al. 2018).

Second, cluster analysis was used to classify children into more-or-less homogeneous groups on the basis of similarities in their neighborhood histories. Specifically, CLARA (Clustering LARge Applications) cluster analysis was applied, which is an extension to the Partitioning Around Medoids (PAM) algorithm, specifically designed to deal with large data sets (for details, see Kaufman and Rousseeuw 1990). CLARA draws multiple samples of the data, applies PAM clustering on each sample, and returns its best clustering as the output. The number of clusters needs to be specified in advance in CLARA. A range of cluster solutions (2–20 cuts) were tested of which the quality was determined with the Average Silhouette Width (ASW) criterion. The 7-cluster solution was found to be optimal (ASW = 0.57). The “Results” section starts off with a detailed description of the clusters.

Finally, in order to examine how cluster membership is related to the three types of problem behavior, a series of binary logistic regression models were estimated. Robust confidence intervals clustered by neighborhoods at age 17 were used to account for the clustering of individuals in neighborhoods. Age 17 was chosen for clustering as the outcome variables are measured around this age (cf. Morris et al. 2018). The analysis concludes with several robustness checks.

## Results

### Typology of Neighborhood Trajectories

**Fig. 2:**
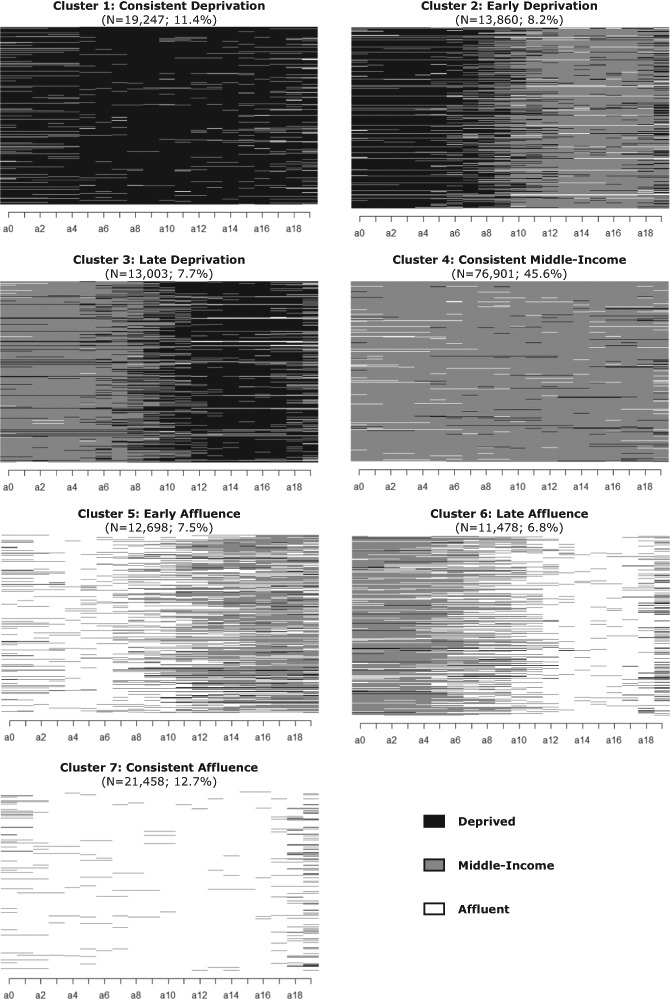
Sequence index plots of seven clusters of children’s neighborhood trajectories

All individual sequences were grouped into seven broader types of neighborhood trajectories. Figure 2 shows the sequence index plots for each of these seven trajectory types. In sequence index plots, each individual is represented by a separate horizontal line that is colored according to the type of neighborhood at each age—black for deprived, gray for middle-income, and white for affluent neighborhoods. We thus visualize the longitudinal succession of neighborhood types for each individual as well as, through the length of each color segment, the duration spent in each neighborhood type. In addition, the medoid sequence (smallest sum of pairwise distances to all other sequences in the group) is reported as the most characteristic sequence within each cluster (for details, see Aassve et al. 2007). The medoid sequences are reported in the so-called state-permanence-sequence format. In this format, each successive distinct state in the sequence is given together with its duration. The following three sequence states are distinguished: D (deprived neighborhood), M (middle-income neighborhood), or A (affluent neighborhood).

Cluster 1 (consistent deprivation) accounts for 11% of the sample and has the medoid sequence [D/20], which stands for a trajectory in which a person has lived in a deprived neighborhood during the complete observation period. The sequences in this cluster are thus characterized by long-term exposure to a deprived neighborhood during childhood. This does not necessarily mean that individuals in this cluster had never changed residences during the observation period, but if they moved, they typically moved from one deprived neighborhood to another. The trajectories in Cluster 2 (early deprivation) are characterized by the medoid sequence [D/6–M/14]. Individuals who experienced such a trajectory were born in a deprived neighborhood and moved towards a middle-income neighborhood at the age of 6 to live there for the remainder of the observation period. Cluster 3 (late deprivation) includes those who followed the opposite path: they were born in middle-income neighborhoods, but moved towards deprived neighborhoods as they grew older. The medoid sequence [M/7–D/13] suggests that the duration of exposure to neighborhood disadvantage is similar to that of children in Cluster 2. Clusters 2 and 3 each cover about 8% of the total sample.

Cluster 4 (consistent middle-income) comprises by far the largest group of the sample (46%) and has the medoid sequence [M/20], reflecting a sequence in which an individual has lived in a middle-income neighborhood throughout the entire childhood life course. Cluster 5 (early affluence), accounting for about 8% of the sample, includes children who were living in an affluent neighborhood during early childhood and in a middle-income neighborhood during adolescence. This is reflected in the medoid sequence [A/6–M/14]. Cluster 6 (late affluence) is the smallest cluster of the sample (7%) and has the medoid sequence [M/8–A/12]. This cluster is thus the opposite of Cluster 5, with exposure to middle-income neighborhoods during early childhood and exposure to neighborhood affluence in adolescence. Finally, Cluster 7 (consistent affluence) is characterized by living in an affluent neighborhood throughout childhood, reflected by the medoid sequence [A/20].

### Neighborhood Trajectories and Problem Behavior

Now that each of the clusters have been described, we examine how they are related to the probability of teenage parenthood, school dropout, and delinquent behavior. Six dummy variables were generated that compare the effect of membership in Clusters 2–7 to the effect of membership in Cluster 1 (consistent deprivation). Table 3 presents the results of a series of binary logistic regression models relating the three outcome variables to these dummy variables. For each dependent variable, two models were estimated. In the first model, (under Model 1) only the dummy variables for the different trajectory types are included. In the next set of models (under Model 2), a range of control variables were added in order to assess the extent to which the neighborhood effects are attributable to other observed characteristics.

Models 1a-c (Table 3) show that children in all clusters, except the late deprivation group, have a significantly lower likelihood to engage in any of the behavioral problems than children who were long exposed to neighborhood deprivation (consistent deprivation). Especially children who had lived in an affluent neighborhood throughout childhood are less likely to engage in any type of problematic behavior. Specifically, the consistent affluence group is 6 times less likely to become a teenage parent (OR = 0.15, 95% CI[0.11–0.21], *p* < .001), twice less likely to drop out of school (OR = 0.53, 95% CI[0.50–0.57], *p* < .001), and 1.5 times less likely to engage in delinquent behavior (OR = 0.66, 95% CI[0.62–0.72], *p* < .001) than the consistent deprivation group (Table 3, models 1).

While children in the early deprivation group are thus less likely to demonstrate problem behavior than the consistent deprivation group, children who only lived in a deprived neighborhood in adolescence (late deprivation group) do not differ significantly from the consistent deprivation group in terms of teenage parenthood and delinquency. This suggests that neighborhood deprivation during adolescence has a stronger influence on problem behavior than neighborhood deprivation early in childhood, highlighting the importance of timing of exposure. Additional analysis (not in table) indeed show that, without accounting for control variables, the late deprivation group is 53 percent more likely to become a teenage parent (OR = 1.53, 95% CI[1.20–1.96], *p* = .001), 42 percent more likely to drop out of school (OR = 1.42, 95% CI[1.30–1.55], *p* < .001), and 21 percent more likely to engage in delinquent behavior (OR = 1.21, 95% CI[1.09–1.35], *p* < .001) than the early deprivation group. Furthermore, although the differences are small, children in the late deprivation group are significantly more likely to drop out of school than those in the consistent deprivation group, suggesting that sequencing of neighborhood deprivation is important as well.

We proceed by describing the results obtained when including the control variables in Models 2a-c in Table 3. Differences in coefficients across Models 1 and 2 were formally tested using the KHB decomposition method (Karlson et al. 2012). All changes in coefficients, except for the late deprivation group, were found to be statistically significant with *p* < .001. Regarding teenage parenthood and school dropout, the effects of the neighborhood trajectory types become substantially attenuated in Models 2a and 2b (except for the late deprivation group), but they remain statistically significant and the direction of the effects remains consistent across the two models. With regard to delinquent behavior, however, the results portray a different picture. In Model 1, children in all groups except the late deprivation group were found to be less likely to engage in delinquent behavior than consistent deprivation group. In Model 2, by contrast, there are no significant differences between the consistent deprivation group and the early deprivation and early affluence group. The effects of membership in the consistent middle-income, late affluence, and consistent affluence group remain significant, but change from negative in Model 1c to positive in Model 2c. Somewhat counterintuitively, we thus find that after controlling for background characteristics, children who had lived in an affluent neighborhood throughout the entirety childhood are 22 percent more likely to engage in delinquent behavior than children who had consistently lived in a deprived neighborhood during childhood (OR = 1.22, 95% CI[1.12–1.33], *p* < .001).

Finally, additional analyses were conducted in which we replicated Models 2 of Table 3, but changed the reference category of the neighborhood trajectory variable. For the sake of brevity, only the results are reported where the early deprivation group was used as the reference category. The results of all other possible comparisons are available upon request. Compared to children in the early deprivation group, children in the late deprivation group were more likely to become a teenage parent (OR = 1.28, 95% CI[1.03–1.63], *p* = .046) and more likely to drop out of school (OR = 1.23, 95% CI[1.12–1.35], *p* < .001), while there was no significant difference between the two groups in terms of delinquent behavior with *p* *>* .05. Children in the consistent affluence group (OR = 0.70, 95% CI[0.50–0.99], *p* = .047) and late affluence group (OR = 0.70, 95% CI[0.52–0.94], *p* = .019) were less likely to become a teenage parent than those in the early deprivation group. Children in the consistent affluence group were also found to be less likely to drop out of school (OR = 0.91, 95% CI[0.84–0.99], *p* = .021), while the late affluence group did not differ from the early deprivation group in this regard with *p* > .05. Children in the consistent affluence group (OR = 1.27, 95% CI[1.16–1.39], *p* < .001) and the late affluence group (OR = 1.17, 95% CI[1.07–1.28], *p* < .001) were more likely to engage in delinquent behavior than those in the early deprivation group. There were no significant differences between the early deprivation group and the consistent middle-income and early affluence group on the three problem behaviors with *p* *>* .05. These results again suggest that children’s neighborhood environment during adolescence has a stronger impact on problem behavior than their neighborhood context earlier in childhood.

### Sensitivity Analyses

To determine the robustness of our findings, a series of alternative models were tested. First, due to the relatively low frequency of teenage parenthood in the data (Table 2), a Firth logistic regression model was estimated (Firth 1993). This model implements a penalized maximum likelihood estimation for reducing potential bias when estimating logistic regression models with rare events (King and Zeng 2001). The results yielded nearly identical odds ratios and confidence intervals to those reported in Table 3, meaning that the rather low prevalence of teenage parenthood does not bias the parameter estimates.

**Table 2:** Descriptive statistics of study variables

Variable	Category	% in each category or mean (SD)
Teenage parenthood	Yes	1.0
School dropout	Yes	11.3
Delinquent behavior	Yes	8.1
Neighborhood trajectory	Cluster 1. Consistent deprivation	11.4
	Cluster 2. Early deprivation	8.2
	Cluster 3. Late deprivation	7.7
	Cluster 4. Consistent middle-income	45.6
	Cluster 5. Early affluence	7.5
	Cluster 6. Late affluence	6.8
	Cluster 7. Consistent affluence	12.7
Ethnicity	Native Dutch	83.8
	Turkish	2.7
	Moroccan	2.9
	Surinamese	2.1
	Antillean	0.7
	Other non-Western	2.9
	Western	5.0
Sex	Male	51.2
Educational level	Pre-vocational	53.0
	Higher general	20.6
	Pre-university	26.4
Father’s educational level	Low	23.3
	High	14.5
	Missing	62.2
Mother’s educational level	Low	26.8
	High	15.1
	Missing	58.1
Father’s labor force participation	Mean (SD)	0.88 (0.24)
Mother’s labor force participation	Mean (SD)	0.68 (0.35)
Household income (logged)	Mean (SD)	7.48 (0.48)
Residential mobility	0 moves	33.4
	1 move	32.1
	2 moves	18.4
	≥ 3 moves	16.1
Household size	Mean (SD)	4.33 (0.99)
Parental union status	Stable union	77.8
	Dissolution	17.2
	Never lived together	3.2
	Started living together	1.8
Age difference with father	Mean (SD)	32.98 (5.05)
Age difference with mother	Mean (SD)	30.34 (4.40)

Percentages may not total 100 due to rounding

Source: System of Social Statistical Datasets (SSD)

**Table 3:** Logistic regression models of cluster membership influencing problem behavior in adolescence: Odds ratios (OR) and 95% confidence intervals (CI)

	Teenage parenthood				School dropout				Delinquent behavior			
	Model 1a		Model 2a		Model 1b		Model 2b		Model 1c		Model 2c	
	OR	[95% CI]	OR	[95% CI]	OR	[95% CI]	OR	[95% CI]	OR	[95% CI]	OR	[95% CI]
*Neighborhood trajectory (ref* *=* *cluster 1. Consistent deprivation)*												
Cluster 2. Early deprivation	0.67***	[0.56–0.80]	0.78*	[0.64–0.95]	0.76***	[0.72–0.81]	0.90**	[0.84–0.97]	0.80***	[0.74–0.86]	0.96	[0.89–1.04]
Cluster 3. Late deprivation	1.03	[0.83–1.27]	1.00	[0.80–1.27]	1.08*	[1.00–1.17]	1.11*	[1.02–1.20]	0.97	[0.88–1.07]	0.99	[0.89–1.10]
Cluster 4. Consistent middle-income	0.46***	[0.41–0.52]	0.78***	[0.68–0.90]	0.68***	[0.65–0.71]	0.94**	[0.89–0.98]	0.79***	[0.75–0.83]	1.09**	[1.03–1.15]
Cluster 5. Early affluence	0.43***	[0.35–0.52]	0.71**	[0.58–0.87]	0.66***	[0.62–0.71]	0.87***	[0.82–0.93]	0.80***	[0.75–0.86]	1.05	[0.97–1.15]
Cluster 6. Late affluence	0.30***	[0.24–0.39]	0.55***	[0.42–0.72]	0.59***	[0.55–0.63]	0.88**	[0.82–0.95]	0.69***	[0.64–0.75]	1.13**	[1.05–1.22]
Cluster 7. Consistent affluence	0.15***	[0.11–0.21]	0.55***	[0.40–0.76]	0.53***	[0.50–0.57]	0.82***	[0.76–0.88]	0.66***	[0.61–0.72]	1.22***	[1.12–1.33]
*Ethnicity (ref* *=* *native Dutch)*												
Turkish			0.27***	[0.18–0.38]			1.18***	[1.08–1.27]			1.27***	[1.15–1.41]
Moroccan			0.51***	[0.38–0.68]			1.39***	[1.27–1.52]			1.94***	[1.76–2.13]
Surinamese			1.67***	[1.31–2.12]			1.11*	[1.00–1.24]			1.39***	[1.24–1.56]
Antillean			2.47***	[1.66–3.69]			1.23*	[1.02–1.49]			1.28*	[1.03–1.59]
Other non-Western			0.76	[0.57–1.02]			1.04	[0.94–1.14]			1.20***	[1.08–1.33]
Western			1.08	[0.86–1.35]			1.17***	[1.09–1.26]			1.21***	[1.11–1.31]
Male			0.19***	[0.17–0.22]			1.46***	[1.41–1.51]			2.69***	[2.58–2.80]
*Educational level (ref* *=* *pre-vocational)*												
Higher general			0.35***	[0.29–0.43]			0.30***	[0.29–0.33]			0.59***	[0.56–0.63]
Pre-university			0.12***	[0.09–0.16]			0.16***	[0.15–0.17]			0.37***	[0.35–0.40]
*Father’s educational level (ref* *=* *low)*												
High			0.83*	[0.68–1.00]			0.91**	[0.85–0.96]			0.94*	[0.88–1.00]
Unknown			0.94	[0.83–1.07]			0.98	[0.94–1.02]			0.98	[0.94–1.02]
*Mother’s educational level (ref* *=* *low)*												
High			1.02	[0.87–1.21]			0.93**	[0.88–0.98]			0.94	[0.89–1.00]
Unknown			0.97	[0.86–1.10]			0.94**	[0.91–0.98]			0.89***	[0.86–0.93]
Father’s labor force participation			0.58***	[0.48–0.71]			0.62***	[0.58–0.66]			0.63***	[0.59–0.69]
Mother’s labor force participation			0.55***	[0.47–0.64]			0.68***	[0.64–0.71]			1.05	[0.99–1.11]
Household income (logged)			0.67***	[0.59–0.77]			0.93**	[0.89–0.97]			0.90***	[0.86–0.95]
*Residential mobility (ref* *=* *0 moves)*												
1 move			2.00***	[1.66–2.41]			1.14***	[1.09–1.19]			1.05*	[1.00–1.10]
2 moves			3.30***	[2.72–3.99]			1.35***	[1.29–1.42]			1.15***	[1.09–1.22]
≥ 3 moves			5.37***	[4.47–6.46]			1.95***	[1.85–2.05]			1.37***	[1.30–1.45]
Household size			1.20***	[1.15–1.26]			0.97***	[0.95–0.99]			0.99	[0.97–1.01]
*Parental union status (ref* *=* *stable union)*												
Dissolution			1.42***	[1.24–1.63]			1.53***	[1.47–1.60]			1.42***	[1.35–1.49]
Never lived together			1.78***	[1.40–2.25]			1.87***	[1.70–2.05]			1.75***	[1.57–1.95]
Started living together			1.25	[0.92–1.69]			1.59***	[1.43–1.77]			1.48***	[1.31–1.68]
Age difference with father			0.98*	[0.97–1.00]			1.00*	[0.99–1.00]			0.99*	[0.99–1.00]
Age difference with mother			0.97**	[0.96–0.99]			0.99***	[0.98–0.99]			0.99*	[0.99–1.00]
Constant	0.02***	[0.02–0.02]	0.72	[0.28–1.84]	0.18***	[0.17–0.18]	0.70*	[0.50–0.98]	0.11***	[0.10–0.11]	0.22***	[0.15–0.32]
Pseudo R^2^	0.02		0.18		0.01		0.10		0.01		0.07	
−2 Log Likelihood	−8883.45	−6971.67	−59050.48	−51113.67	−47461.15	−42674.27						

The 95% confidence intervals have been corrected for clustering of individuals in neighborhoods at age 17

Source: System of Social Statistical Datasets (SSD)

*p < .05, **p < .01, ***p < .001

Second, due to the relatively large gender differences in the likelihood of the different types of problem behavior, we repeated the regression analysis of Table 3 (models 2) for men and women separately. Among men, the difference in the likelihood of teenage parenthood between the consistent deprivation group and the early deprivation group was not statistically significant after controlling for confounding factors (OR = 0.86, 95% CI[0.55–1.36], *p* = .531). Among women, there were no significant differences between the consistent middle-income group and the consistent deprivation group in school dropout (OR = 0.94, 95% CI[0.88–1.01], *p* = .112) and delinquent behavior (OR = 1.05, 95% CI[0.97–1.15], *p* = .227) after the inclusion of control variables. Other than that, there were no notable differences between the pooled and gender-specific models.

Third, the decision to define neighborhoods in the top 20 percent of the income distribution as “affluent” and those in the bottom 20 percent as “deprived” is to a certain extent arbitrary. Therefore a sensitivity analysis was conducted using deciles rather than quintiles of neighborhood income. The research population was divided into three groups: children who lived for 15–20 years in the bottom 10 percent of the neighborhood income distribution (4.5%), children who lived for 15–20 years in the top 10 percent of the neighborhood income distribution (5.3%), and children who did not meet these criteria (90.2%). Compared to children who were long exposed to the poorest decile of neighborhood income, children who were long exposed to the most affluent decile of neighborhood income were less likely to become a teenage parent (OR = 0.56, 95% CI[0.35–0.90], *p* = .017), less likely to drop out of school (OR = 0.66, 95% CI[0.60–0.74], *p* < .001), and more likely to engage in delinquent behavior (OR = 1.23, 95% CI[1.09–1.39], *p* = .001) after accounting for control variables. These findings are similar to the differences between the consistent deprivation and consistent affluence group in Table 3 (models 2), meaning that our findings are robust to different cut-off points for neighborhood income.

Fourth and lastly, not every individual that engaged in problem behavior did so specifically at age 19 (Table 1). This could be problematic because, for some children, neighborhood exposure thus partly occurred later in time than the dependent variable. Therefore, a 7-cluster typology was also constructed using the following age ranges: 0–17, 0–15, and 0–12. The clusters based on the complete age range (0–19) and the restricted age ranges were found to overlap substantially, with respectively 97, 92, and 86 percent being grouped in the same cluster. Thus, if we would model children’s neighborhood trajectories up to occurrence of problem behavior, the typology would be very similar to the current classification.

## Discussion

There is a persuasive theoretical basis for the view that neighborhood effects on children’s life chances depend on duration, timing, and sequencing of neighborhood deprivation during childhood (Sharkey and Faber 2014). However, empirical studies addressing the temporal aspects of neighborhood effects have predominantly only focused on how long children have been exposed to neighborhood disadvantage over their childhood (duration). As such, research on the impact of exposure to neighborhood disadvantage at different stages in childhood (timing) and changes in children’s neighborhood circumstances over time (sequencing) is still limited and, moreover, does not yield consistent results. In this study, sequence analysis was applied to simultaneously capture children’s duration, timing, and sequencing of exposure to poor and nonpoor neighborhoods during childhood, providing a much more comprehensive measure of their neighborhood experiences.

The sequence analysis identified seven substantively different types of neighborhood trajectories in childhood. In three of these types, children had lived in a deprived neighborhood at some point during childhood, but differed in terms of duration, timing, and sequencing of exposure. Some children experienced neighborhood disadvantage throughout childhood (consistent deprivation), while other children were exposed to a deprived neighborhood either only early in childhood (early deprivation) or only during adolescence (late deprivation). In three of the four remaining trajectory types in the classification, children had lived in an affluent neighborhood with a similar threefold distinction in terms of patterns of exposure: throughout childhood (consistent affluence), only early in childhood (early affluence), or only during adolescence (late affluence). The last remaining trajectory type included children who had lived in a middle-income neighborhood throughout childhood (consistent middle-income).

The next step in the analysis was to examine the extent to which the identified trajectory types were related to three types of behavioral problems in adolescence: teenage parenthood, school dropout, and delinquent behavior. Children who had consistently lived in a deprived neighborhood during childhood (i.e., consistent deprivation group) were found to be more likely to become a teenage parent and/or to drop out of school than children who were exposed to more advantaged neighborhood circumstances. The only exception here were children who had lived in a deprived neighborhood only in adolescence (i.e., late deprivation group)—they did not differ from the consistent deprivation group in terms of teenage parenthood and were even more likely to drop out of school. These results first of all underscore the importance of adolescent exposure to neighborhood disadvantage on subsequent problem behavior (Wodtke et al. 2016). The finding that the late deprivation group was more likely to drop out of school than the consistent deprivation group suggests that the sequencing of neighborhood deprivation is important as well. The finding is in line with previous research on family poverty showing that children whose family income declines are at greater risk of problem behavior than children who experience stable but disadvantaged economic circumstances (Moore et al. 2002).

Our findings with regard to delinquent behavior were interestingly different from those discussed above. Whereas the direction of effects of the trajectory types was similar to that for teenage parenthood and school dropout on the bivariate level, the results changed substantially after accounting for various individual and parental background characteristics. More precisely, after holding all control variables constant, particularly children who were exposed to neighborhood affluence throughout childhood (i.e., consistent affluence group) and those exposed to an affluent neighborhood only in adolescence (i.e., late affluence group) were most likely to engage in delinquent behavior. A possible explanation for this somewhat counterintuitive finding may be that there are higher levels of community surveillance and more frequent reporting of suspicious behaviors to the police in more affluent neighborhoods (Varano et al. 2009). Another explanation could be that unobserved characteristics of the children’s parents make the parents in affluent neighborhoods more likely to report their child(ren) to the police if they realise or suspect any signs of criminality. Furthermore, although measured at the family rather than the neighborhood level, previous research has shown that affluent, high-achieving youth are statistically more likely than normative samples to show serious disturbance across several domains including drug and alcohol use, as well as internalizing and externalizing problems (for a review, see Luthar et al. 2013). Various explanations have been proposed for this, including perceived criticism by parents, peer envy, and negative peer interactions (Coren and Luthar 2014). Future research may more specifically test these conjectures.

The sequence analysis approach delivered results that would not have been uncovered by more conventional approaches for assessing children’s exposure to neighborhood deprivation, such as single point-in-time and cumulative measures of exposure. Nevertheless, this study suggest that point-in-time measures of neighborhood quality introduce only limited bias in the analyses as long as they are measured during adolescence. Indeed, point-in-time measures of neighborhood quality in adolescence conflate the effect of long-term neighborhood advantage or disadvantage in childhood (i.e., consistent affluence/deprivation) with the effect of more recent exposure to neighborhood affluence or deprivation (i.e., late affluence/deprivation). However, this may be less problematic than previously thought, because we hardly find differences between consistent and adolescent exposure to neighborhood (dis)advantage. Even so, longitudinal measures are to be preferred because they are less sensitive to random noise or transitory fluctuations in children’s neighborhood status. Moreover, while this study found no noteworthy differences in the effects of consistent and adolescent exposure to neighborhood deprivation on adolescent problem behavior, such differences may well exist for other outcomes of children’s lives, such as cognitive development and (psychological) health.

It is worth noting, furthermore, that this study focused on the Netherlands and the findings may not be generalizable beyond the Dutch context. Regarding the independent variable, it is important to mention that urban problems have received ample policy attention in the Netherlands over the past decades. Various area-based policies have been developed specifically directed at countering residential segregation and the spatial concentration of low-income households (Kleinhans 2004). Consequently, poor neighborhoods in the Netherlands may be relatively affluent compared to poor neighborhoods in other countries. Regarding the dependent variables, we found a much lower prevalence of teenage parenthood than previous studies in the US (Wodtke 2013), which may be related to country differences in laws and regulations regarding abortion and/or norms about sexuality and childbearing. Additionally, the legalization, availability, and costs of contraception differ across countries as well and are strongly related to the likelihood of teenage pregnancy (Levels et al. 2010). The findings regarding delinquency may also be difficult to generalize to another country context, because the Dutch Bureau Halt programme does not exist in other countries. Thus, research testing the influence of duration, timing, and sequencing of neighborhood (dis)advantage in childhood on adolescent problem behavior in other countries is clearly warranted and called for.

Finally, this study has several limitations. Especially regarding delinquent behavior, it is important to bear in mind that this study used administrative data which obviously only provide information on registered delinquency. It is well known that a large proportion of crimes are not reported to the police (and thus remain unregistered), especially so with regard to minor offences. More importantly, reporting practices have been found to vary across different types of neighborhoods (Varano et al. 2009). Furthermore, because in some cases the identity of the biological father may be unknown, registration effects may also play a role in the accuracy of our measure of teenage fatherhood, but it is unlikely that this has seriously biased the results. Another limitation of this study is that the data lack information on several potentially important variables, such as such as parenting styles and cultural values. Consequently, we are unable to fully account for the self-selection of parents into specific types of neighborhoods, which may partly explain the observed associations. Finally, although sequence analysis provides a much more complete measure of children’s experiences of neighborhood deprivation than other approaches, it does not allow for the inclusion of time-varying variables. Time-varying covariates such as household income and parental employment status influence selection into different neighborhoods, but may themselves also be affected by past neighborhood conditions (Wodtke 2013). We cannot take into account these dynamics using sequence analysis, which possibly leads to an underestimation of neighborhood effects. Yet, the underestimation is unlikely be very large as previous research suggests that neighborhood effects on individual socioeconomic outcomes are relatively small (Miltenburg 2017).

## Conclusion

This study highlights the need to consider different aspects of children’s exposure to neighborhood deprivation during their childhood. Whereas previous research showed that children exposed to poor neighborhoods for extended periods have worse outcomes than children exposed to neighborhood poverty for only a short time (e.g., Wodtke et al. 2011), the current study showed that it is also crucially important *when* in childhood the exposure occurs, i.e. the timing of exposure. More precisely, children who were exposed to neighborhood deprivation only during adolescence were found to be equally likely to become a teenage parent and to engage in delinquent behavior as children who spent the entirety of their childhood in a deprived neighborhood. Compared to children who lived in a deprived neighborhood only during early childhood, children who lived in a deprived neighborhood only during adolescence were more likely to become a teenage parent and to drop out of school. These findings indicate the importance of exposure to neighborhood disadvantage across different developmental stages in childhood, pointing to adolescence as the most crucial period. Although our findings did not provide strong evidence for the importance of sequencing of exposure, children who whose neighborhood status declined during childhood (i.e., late deprivation group) were found to be slightly more likely to drop out of school than those who experienced stable but disadvantaged neighborhood circumstances (i.e., consistent deprivation group). All in all, these findings highlight the importance of simultaneously taking into account children’s duration, timing, and sequencing of exposure to neighborhood deprivation.

This study is a first step in class-based trajectory modelling of neighborhood exposures to predict outcome variables. Future research may elaborate on this study by using other data sets and/or outcome variables. For example, using the Swedish register data which go back further in time, future studies can track individual neighborhood trajectories from birth all the way up to middle adulthood and identify patterns of exposure to neighborhood (dis)advantage for more stages across the human life span. Another potentially interesting avenue for future research would be to use multichannel sequence analysis to simultaneously take into account the duration, timing, and sequencing of exposure to disadvantage in multiple spheres of life, such as neighborhoods, schools, and households. This is a complex task for further research.

## Footnotes

1 Children who died or emigrated during the observation period are excluded from the analysis so that all neighborhood trajectories are of equal length. Comparing sequences of different length is problematic because the length of sequences has itself an impact on the analyses (Billari, 2001). Children whose both parents died during the observation period are excluded from the analysis because they often reside in institutional households afterwards (e.g., orphanages) for which address information was not available.

2 Because income data were not available for the years earlier than 1999, we use the average neighborhood income in 1999 to determine the quality of the neighborhoods for the period 1995-1998. Previous research has shown that neighborhood change is a slow process (Zwiers et al., 2016), implying that this should introduce only limited bias in the analyses.
